# Supplementary material for: Neutralizing antibodies correlate with protection from SARS-CoV-2 in humans during a fishery vessel outbreak with high attack rate
Source: medRxiv. 2020 Aug 14:2020.08.13.20173161. Preprint. [Version 1] doi: 10.1101/2020.08.13.20173161 (PMC7430625; doi:10.1101/2020.08.13.20173161)
Supplement: 1 [file NIHPP2020.08.13.20173161-supplement-1.pdf]

medRxiv preprint doi: https://doi.org/10.1101/2020.08.13.20173161.this version posted August 14, 2020. The copyright holder for this preprint
(which was not certified by peer review) is the author/funder, who has granted medRxiv a license to display the preprint in perpetuity.
It is made available under a CC-BY 4.0 International license .

Supplemental Table 1 – SARS-CoV-2 isolates and accessions sequenced in this study.

Isolate	GISAID Accession Number
USA/WA-UW-10027/2020	EPI_ISL_461450
USA/WA-UW-10028/2020	EPI_ISL_461451
USA/WA-UW-10029/2020	EPI_ISL_461452
USA/WA-UW-10030/2020	EPI_ISL_511852
USA/WA-UW-10031/2020	EPI_ISL_461453
USA/WA-UW-10034/2020	EPI_ISL_511853
USA/WA-UW-10036/2020	EPI_ISL_461454
USA/WA-UW-10038/2020	EPI_ISL_511854
USA/WA-UW-10039/2020	EPI_ISL_461455
USA/WA-UW-10040/2020	EPI_ISL_461456
USA/WA-UW-10042/2020	EPI_ISL_461457
USA/WA-UW-10088/2020	EPI_ISL_461458
USA/WA-UW-10089/2020	EPI_ISL_461459
USA/WA-UW-10090/2020	EPI_ISL_461460
USA/WA-UW-10091/2020	EPI_ISL_461461
USA/WA-UW-10093/2020	EPI_ISL_461462
USA/WA-UW-10094/2020	EPI_ISL_461463
USA/WA-UW-10101/2020	EPI_ISL_511855
USA/WA-UW-10102/2020	EPI_ISL_461464
USA/WA-UW-10105/2020	EPI_ISL_511856
USA/WA-UW-10106/2020	EPI_ISL_461465
USA/WA-UW-10107/2020	EPI_ISL_461466
USA/WA-UW-10108/2020	EPI_ISL_461467
USA/WA-UW-10113/2020	EPI_ISL_511857
USA/WA-UW-10114/2020	EPI_ISL_461468
USA/WA-UW-10115/2020	EPI_ISL_511858
USA/WA-UW-10116/2020	EPI_ISL_512086
USA/WA-UW-10117/2020	EPI_ISL_461469
USA/WA-UW-10118/2020	EPI_ISL_461470
USA/WA-UW-10124/2020	EPI_ISL_511859
USA/WA-UW-10126/2020	EPI_ISL_511860
USA/WA-UW-10127/2020	EPI_ISL_461471
USA/WA-UW-10128/2020	EPI_ISL_461472
USA/WA-UW-10129/2020	EPI_ISL_461473
USA/WA-UW-10130/2020	EPI_ISL_461474
USA/WA-UW-10131/2020	EPI_ISL_461475
USA/WA-UW-10133/2020	EPI_ISL_511861
USA/WA-UW-10136/2020	EPI_ISL_461476
USA/WA-UW-10138/2020	EPI_ISL_461477
